# Primary malignant melanoma of the cervix: A case report

**DOI:** 10.3892/ol.2014.2555

**Published:** 2014-09-24

**Authors:** ZHUQING LIU, HUI WANG, XI ZHANG, QING XU

**Affiliations:** Department of Medical Oncology, Shanghai Tenth People’s Hospital, Tongji University, School of Medicine, Shanghai 200072, P.R. China

**Keywords:** primary malignant melanoma, cervix

## Abstract

Primary malignant melanoma (MM) of the uterine cervix is a rare neoplasm and the overall prognosis of patients with this disease is poor. Immunohistochemical methods and exclusion of other primary melanoma sites are used to confirm the diagnosis. In the present study, the case of a 65-year-old female patient with an MM of the uterine cervix is reported. Diagnosis was confirmed by immunohistochemical methods using human melanoma black 45 antibody and S-100 protein. The tumor was identified as stage IB1 using the International Federation of Gynecology and Obstetrics classification. Chest X-ray and abdominopelvic computed tomography results were normal. The patient subsequently underwent a radical hysterectomy with bilateral salpingo-oophorectomy and pelvic lymphadenectomy. Following combined radical surgery and chemotherapy, complete remission of the tumor was achieved. The patient has since been well for 30 months without recurrence subsequent to the surgery.

## Introduction

Malignant melanoma (MM), a common neoplasm of the skin and mucous membranes, constitutes 1% of all cancer cases (1). In total, 5% of melanocytic malignancies in females occur in the vulva, with rare cases detected in the ovary, uterus or uterine cervix (2). MM commonly clinically presents at an advanced stage, and the diagnosis is confirmed by histological examination using certain staining techniques and by immunohistochemical analysis (3). The majority of patients respond poorly to therapy (4). Certain therapeutic regimens are recommended for cervical melanoma, including radical hysterectomy with pelvic lymph node dissection, and partial vaginectomy followed by radiation therapy with either intracavitary or external beam radiation, or the two treatments combined. However, the majority of patients exhibit poor long-term survival (5). Written informed consent was obtained from the patient.

## Case report

In June 2011, a female who presented with a one-week history of bleeding from the vagina was admitted to the Shanghai Tenth People’s Hospital (Shanghai, China). A gynecological examination revealed a ulcero-proliferative lesion that measured 1×2.5 cm^2^ on the anterior lip of the cervix. The parametrium was not involved and the growth was primarily restricted to the cervix. An incisional biopsy was performed and the sample sent for histopathological examination. The lesion histopathology revealed a malignant neoplasm, which indicated the possibility of a primary uterine cervical melanoma. The chest X-ray and abdominopelvic computed tomography results were normal. The patient was administered chemotherapy consisting of 20 mg/m^2^ intravenous cisplatin, 250 mg/m^2^ dacarbazine and 1 mg/m^2^ vincristine in each cycle, and received two such cycles at four week intervals.

**Figure 1:**
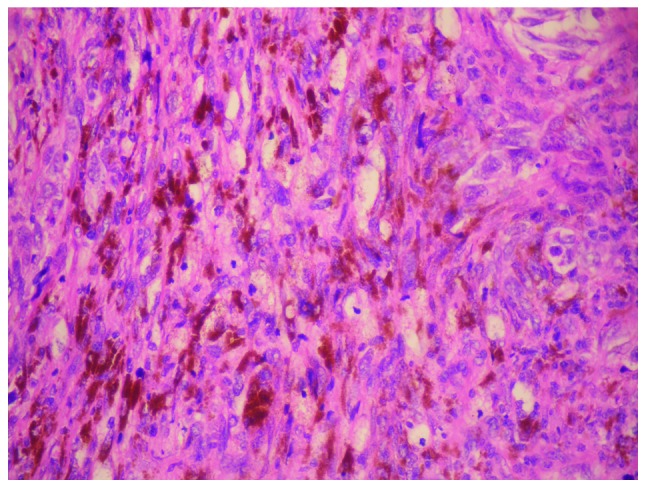
Immunohistochemical image of the cervical malignant melanoma specimen. Hematoxylin and eosin stain; magnification, ×400.

**Figure 2:**
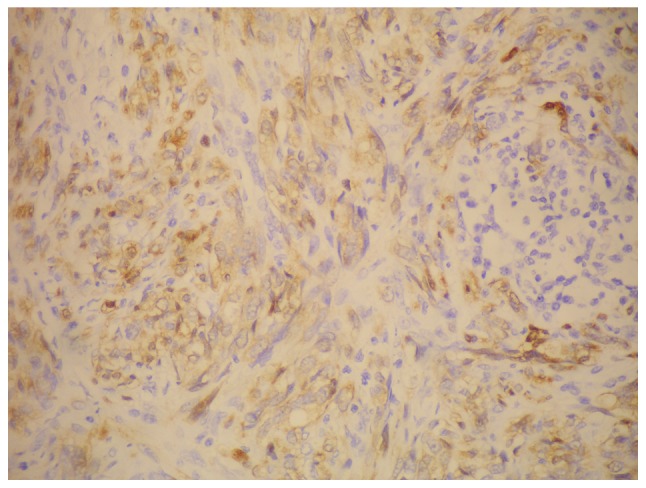
Immunohistochemical image of S100 protein expression in the cervical malignant melanoma sample. Magnification, ×400.

**Figure 3:**
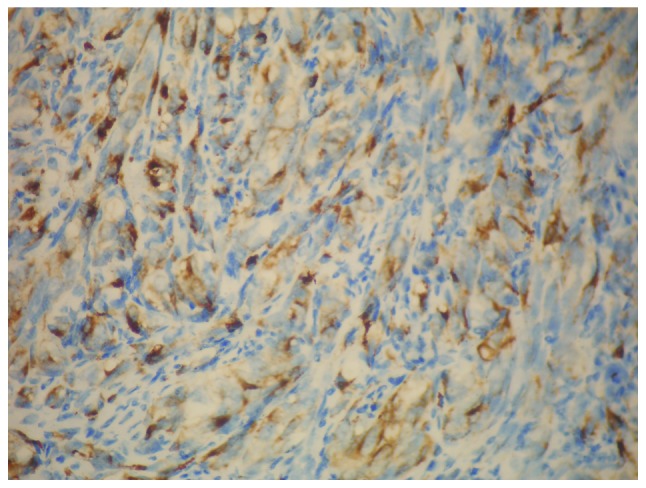
Immunohistochemical image of human melanoma black 45 expression in the cervical malignant melanoma specimen. Magnification, ×400.

**Figure 4:**
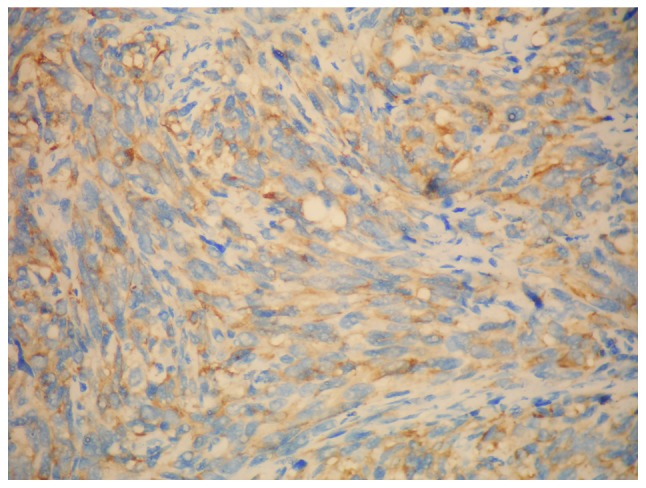
Immunohistochemical image of melanoma antigen recognized by T-cells expression in the cervical malignant melanoma sample. Magnification, ×400.

The patient then underwent radical hysterectomy with bilateral salpingo-oophorectomy and pelvic lymphadenectomy. A specimen of the uterus, cervix and vaginal cuff with bilateral attached adnexae, and a pelvic lymph node dissection specimen were received for analysis. Gross examination revealed a 4×2.5-cm^2^ ulcero-proliferative lesion in the cervix. The microscopic features of the specimen were comparable with those observed in the pre-operative biopsy (Fig. 1). The final diagnosis for the patient was International Federation of Gynecology and Obstetrics stage IB1 melanoma of the cervix with no lymph node metastasis. Immunohistochemistry revealed diffuse positive reactions for S-100 protein (Fig. 2), human melanoma black 45 (HMB-45; Fig. 3) and melanoma antigen recognized by T-cells (Fig. 4), which confirmed the diagnosis of MM, with no reaction for epithelial markers, namely high molecular weight-cytokeratin and epithelial membrane antigen.

A comprehensive assessment for melanotic lesions in the uveal tract (opthalmoscopy)*,* skin and other mucosal sites was negative. A pre-operative abdominal ultrasound examination revealed that the structures of the spleen, liver, bowel, kidneys and retroperitoneum were normal. Since a primary melanoma and epitheliotropism of the melanoma cells was not present, a diagnosis of primary cervical melanoma was reached. Subsequently, the patient received one cycle of combination chemotherapy with dacarbazine, cisplatin and vincristine. This regimen was followed by chemotherapy using 20 mg/m^2^ intravenous cisplatin and 250 mg/m^2^ dacarbazine between days 1 and 3 in each cycle, for four such cycles at four week intervals. The final chemotherapy cycle was administered in January 2012. The patient was kept under close observation and has been well for 30 months without recurrence subsequent to surgery. The most recent imaging results, including chest X-ray and abdominopelvic computed tomography results, were normal. The patient has subsequently had regular follow-up assessments.

## Discussion

Primary melanoma of the cervix is a rare condition. A total of 5% melanocytic malignancies in females occur in the vulva, with rare malignancies also detected in the ovary, uterus and uterine cervix (2). Cervical melanoma is hypothesized to arise from the cervical melanocytic cells. The entire spectrum of melanocytic lesions, including blue nevi, benign lentigines and melanomas, are known to occur in the cervix (4). As the peak incidence of primary carcinoma of the uterine cervix occurs between 40 and 50 years of age, this places the patient in the present case study, aged 65 years, in an older age group, although whether prognosis depends on the age of the patient is not known.

Diagnosis of cervical MM is usually determined by gynecological examination, histopathology and electron microscopy, and is confirmed by immunohistochemical staining with S-100 and HMB-45. As the cervix was not previously considered to contain melanocytes, the possibility of cervical melanoma was controversial for a long period of time. Therefore, ruling out the diagnosis of melanoma elsewhere in the body prior to the diagnosis of primary cervical melanoma is important. Furthermore, the cervix is not a common site for metastatic malignancies, due to a limited blood supply and the poor nature of the cervical fibrous stroma site for tumor growth. Morris and Taylor (6) suggested the following diagnostic criteria: (i) The presence of melanin in the normal cervical epithelium; (ii) the absence of melanoma anywhere else in the body; (iii) the demonstration of junctional change in the cervix; and (iv) metastases according to the pattern of cervical carcinoma.

Little consensus has been achieved with regard to what is the most effective approach in terms of the clinical management of MM of the uterine cervix. Radical hysterectomy is the most common procedure (5,7–9). The recommended treatment is usually surgery, including radical hysterectomy, pelvic lymphadenectomy and partial vaginectomy (10). Although the benefit of lymphadenectomy is controversial, this treatment should be advocated in cases with large growth of the tumor and the presence of pigmented lymph nodes, which signifies a higher risk of secondary metastases. Post-operative chemotherapy is a further viable treatment option in these cases, as numerous patients develop metastatic disease (11). Dacarbazine, a drug that has been demonstrated to reduce tumor size in patients with cutaneous MM, may have potential in cervical MM treatment (11). In the present case study, a combination of dacarbazine, cisplatin and vincristine was advocated, with which the patient remained disease-free for a minimum of 29 months following surgery.

The prognosis of primary cervical MM is usually poor. Survival times are variable, but usually range between 0.1 month and 14 years. Studies have indicated that 87.5% patients succumb to the disease within three years of diagnosis (10). In one study of 78 patients, although the majority of patients were diagnosed at an early stage, only two survived >5 years (10).

In conclusion, cytopathologists should be aware of this type of lesion upon evaluation of cervical smear cytology in order to provide an early diagnosis of cervical MM. Radical hysterectomy with bilateral salpingo-oophorectomy and pelvic lymphadenectomy is advisable for surgical treatment. Chemotherapy may be also considered in patients following surgery.
